# Efficacy of low-load resistance training combined with blood flow restriction vs. high-load resistance training on sarcopenia among community-dwelling older Chinese people: study protocol for a 3-arm randomized controlled trial

**DOI:** 10.1186/s13063-021-05495-z

**Published:** 2021-08-04

**Authors:** Nan Chen, Xiangfeng He, Guoyun Zhao, Linqian Lu, Barbara E. Ainsworth, Yu Liu, Xie Wu

**Affiliations:** 1 School of Kinesiology, Shanghai University of Sport, Shanghai, 200438 China; 2 Department of Rehabilitation, Xinhua Hospital Chongming Branch, Shanghai, China; 3 Department of Rehabilitation, Xinhua Hospital Affiliated to Shanghai Jiaotong University School of Medicine, Shanghai, China; 4 College of Health Solutions, Arizona State University, Phoenix, AZ USA

**Keywords:** Sarcopenia, Resistance training, Blood flow restriction

## Abstract

**Background:**

Sarcopenia is accompanied by a decline in muscle mass, muscle strength, and muscle function. Resistance training is the most potential training method for the prevention and treatment of sarcopenia. However, the conventional high-load resistance training (CRT) recommended by the American College of Sports Medicine is a challenge for older people with sarcopenia. As a novel training method, low-load resistance training combined with blood flow restriction (LRT-BFR) may elicit similar muscle mass and muscle strength gains as CRT but with less effort. The objectives of this study are to assess and compare the efficacy and safety of 12-week LRT-BFR and CRT on muscle strength, muscle performance, body composition, pulmonary function, blood biomarkers, CVD risk factors, and quality of life in community-dwelling older Chinese people with sarcopenia.

**Method:**

This is a 12-week, assessor-blinded, 3-arm randomized controlled trial with a non-exercise control group. Community-dwelling people over 65 years will be screened for sarcopenia according to the diagnostic criteria of the Asian Working Group for Sarcopenia (AWGS). Fifty-one subjects will be randomized into a LRT-BFR group (*n* = 17), a CRT group (*n* = 17), and a no-strength training control group (*n* = 17). The primary outcome is lower limb muscle strength. The secondary outcomes are body composition, upper limb muscle strength, pulmonary function, blood biomarkers, CVD risk factors, and quality of life. Post-intervention follow-up will be performed for 12 weeks. These indicators will be assessed at baseline (0 week), after the 12-week intervention (12 weeks), and at follow-up (24 weeks). The adverse events will also be reported. Data will be analyzed for all participants in an intent-to-treat plan.

**Discussion:**

This study is the first RCT that will systematically measure and compare the efficacy and safety of LRT-BFR and CRT in older people with sarcopenia on muscle strength, body composition, pulmonary function, blood biomarkers (inflammatory biomarkers, hormone, and growth factors), CVD risk factors, and quality of life. This study can provide an efficient and safe method to prevent the progression of sarcopenia in older people.

**Trial registration:**

Chinese Clinical Trial Registry ChiCTR2100042803. Registered on 28 January 2021.

**Supplementary Information:**

The online version contains supplementary material available at 10.1186/s13063-021-05495-z.

## Background

Sarcopenia is a condition characterized by a progressive decline in skeletal muscle mass, muscle strength, and physical performance associated with advancing age [1]. In 2017, the Asian Working Group for Sarcopenia (AWGS) reported that the prevalence of sarcopenia in persons aged ≥ 40 years ranged from 5.5 to 25.7% in Asian countries [1]. This is consistent with a prevalence of sarcopenia of 18.5% reported by Hu et al. [2] in 607 Chinese community-dwelling older people aged 60–90 years. Sarcopenia is concerning as it causes negative health outcomes to include falls, fractures, physical disabilities, decreased quality of life, and increased premature mortality [3]. As pharmaceutical treatments for sarcopenia are largely ineffective [4], these outcomes increase healthcare costs and bring a heavy economic burden to individuals and societies [5].

Sarcopenia results from complex and interdependent pathophysiological mechanisms. It involves not only muscle tissue loss and muscle contractile dysfunction, but also endocrine and metabolic abnormalities and is related to a low-inflammatory state referred to as inflamm-aging [6]. The mechanisms of sarcopenia are generally unknown; however, changes in specific biomarkers are speculated that characterize the condition. The most common biomarkers are inflammatory biomarkers (such as interleukin-6 [IL-6], tumor necrosis factor alpha [TNF-α], and serum C-reactive protein [CRP]), hormone (such as growth hormone [GH] and insulin-like growth factor 1 [IGF-1]), and growth factors (such as myostatin [MSTN] and follistatin [FST]).

Previous studies have shown that exercise (especially resistance training) is the most effective non-pharmacological intervention for increasing muscle mass and muscle strength. It also preserves muscle performance throughout the aging process [7–9]. To elicit muscle hypertrophy and muscle strength gains, the American College of Sports Medicine (ACSM) suggests older people engage in high-load resistance training programs (60–80% of one-repetition maximum [1RM]) for 2–3 days per week [7]. In addition, a meta-analysis evaluating strength training studies in older people has demonstrated that the effects of low-load resistance training (≤ 60% 1RM) on increasing muscle strength are less effective than those of high-load resistance training (≥ 60% 1RM) [10]. However, high-load resistance training is often perceived as being too difficult and technique-intensive for older people in clinical settings [11], especially frail people with sarcopenia. In addition, high-load resistance training can produce high mechanical stress on joints which may increase the risk of injury in older people [12]. Not researchers agree that high-load resistance training poses a risk for older adults [13, 14]. Nevertheless, in an epidemiological study, Kerr et al. [15] reported that over 90% of strength training injuries in people presenting to emergency departments between 1990 and 2007 were associated with high-load resistance training. Furthermore, the incidence of injuries was positively associated with the decrease in age-associated muscle strength, especially in women. In addition, musculoskeletal disorders in the aging population (such as osteoarthritis, gout, back pain) can decrease muscle strength and/or joint stability, leading to an inability to safely perform heavy load training [16]. Therefore, it is important to find a safe and feasible training method to increase muscle mass and muscle strength and avoid the risk of injury in older people with sarcopenia.

Low-load resistance training (LRT) combined with blood flow restriction (BFR) is a method of stimulating muscle growth by exerting external pressure on the proximal limbs through a compression device, such as a tourniquet or an inflatable cuff, that occludes venous blood flow during resistance training [17, 18]. Recently, several studies have demonstrated that low-load resistance training (20–30% 1RM) combined with BFR (LRT-BFR) may produce the same gains on muscle hypertrophy and muscle strength as observed with conventional high-load resistance training (CRT) [19, 20]. The studies also have demonstrated that LRT-BFR can increase muscle strength in lower [21] and upper limbs [22]; increase muscle size to include muscle cross-sectional area [CSA] [23], muscle thickness, and muscle mass [21, 22]; improve muscle performance evaluated by the timed up and go [TUG] test [23, 24]; decrease fat CSA [21]; and improve quality of life as reported by the 36-item Short-Form Health Survey [SF-36] [23] in older people. Moreover, LRT-BFR may make it easier for novice weight trainers to complete resistance training programs as LRT-BFR does not cause high joint force, subsequently reducing the risks of injury associated with CRT [25].

LRT-BFR may be an alternative resistance training method for healthy older people who are frail, have sarcopenia, or are unable to perform CRT. But some studies indicated that sarcopenia is a correlated risk factor for hyperlipidemia and insulin resistance, which is highly correlated with the risk of cardiovascular disease (CVD) [26]. A cohort with an average follow-up of 11.3 years demonstrated that sarcopenia is associated with higher CVD mortality [27]. Many studies have highlighted the lack of evidence relatively the cardiovascular safety of LRT-BFR particularly in older people at a high risk of CVD [28]. Due to the concerns about LRT-BFR in older people with cardiovascular disease, the efficacy and safety of LRT-BFR in older people diagnosed with sarcopenia should be carefully monitored. Little is known about changes in sarcopenia biomarkers following resistance training in people with sarcopenia. If LRT-BFR is to become a widely used training method for the management of sarcopenia, it is necessary to understand the physiological plus clinical impacts and the potential for cardiovascular adverse outcomes which may result from LRT-BFR training.

As CRT is currently the conventional method to increase muscle strength, size, and performance in older people, it is important to compare the efficacy and safety of LRT-BFR with CRT and a non-exercise comparison group in older people with sarcopenia. Thus, the main aim of this study is to assess and compare the efficacy and safety of two different resistance training interventions (LRT-BFR vs. CRT) with a non-exercising control group on sarcopenia. Thus, the objectives of this study are to (1) systematically evaluate the efficacy of 12-week LRT-BFR and CRT on lower body muscle strength (primary outcome), body composition, muscle performance, pulmonary function, blood biomarkers, CVD risk factors, and quality of life (secondary outcomes) and (2) compare the efficacy and safety between LRT-BFR and CRT, LRT-BFR and CRT, and CRT and no-strength training group, to provide an effective and safe method for the management of sarcopenia.

## Methods

### Study setting

**Fig. 1:**
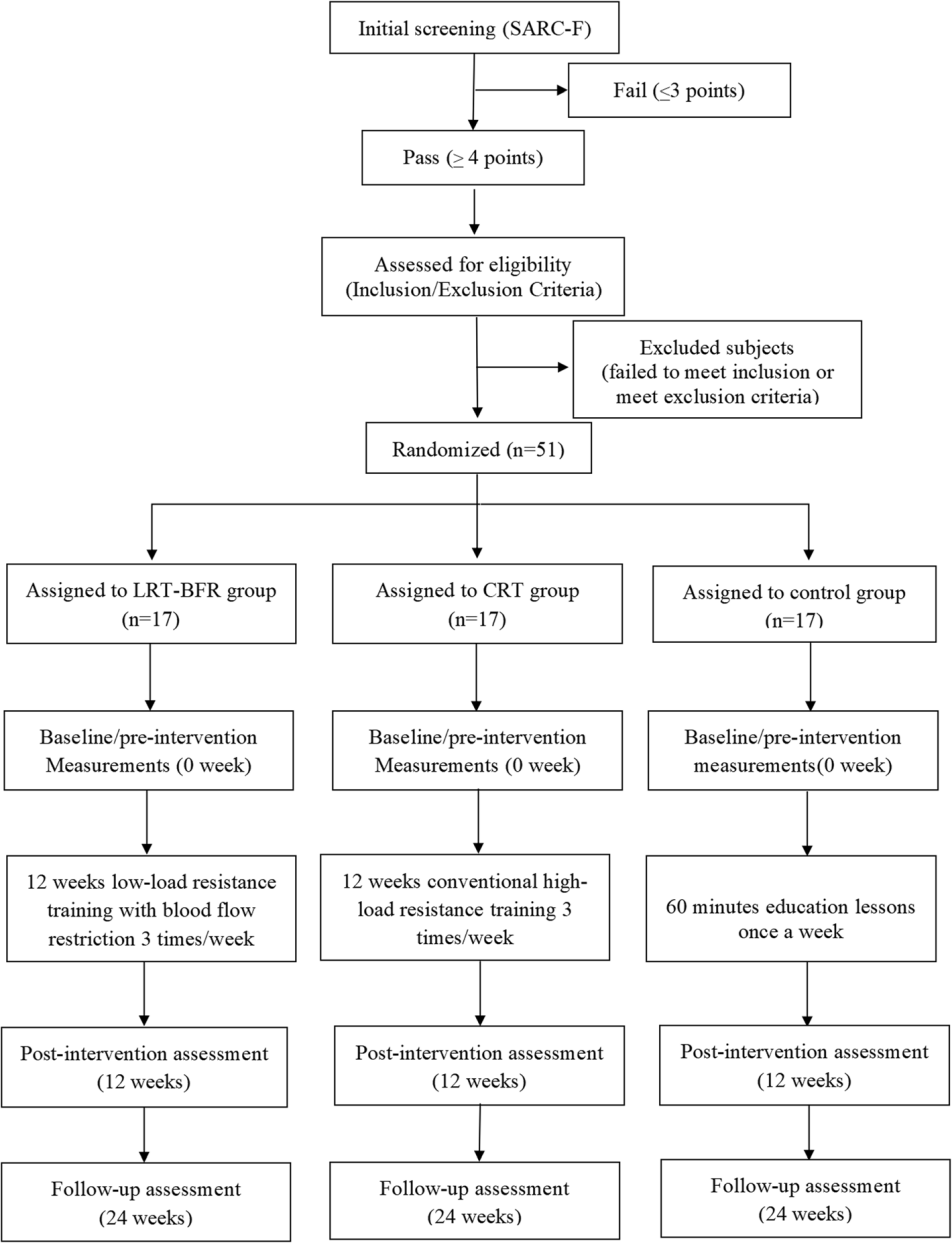
The experimental flow chart showing the patient recruitment, intervention and assessment process

This is an assessor-blinded, 3-arm, randomized intervention-control trial. Older people from four communities in the Chongming District of Shanghai, China, will be screened for the inclusion and exclusion criteria. A total of 51 older people meeting eligibility criteria will be enrolled in the study and randomized into one of three groups (17 in each group): a low-load resistance training with BFR group (LRT-BFR), a conventional high-load resistance training (CRT) group, or an education control group. Baseline, endpoint trial, and follow-up assessments will be conducted. The experimental flow chart is shown in Fig. 1. The Standard Protocol Items: Recommendations for Interventional Trials (SPIRIT) 2013 checklist is shown in Additional file 1. This study will be conducted in March 2021 at Xinhua Hospital Chongming Branch. The enrolment process, intervention, and assessments are presented in Table 1.

**Table 1:** Schedule of enrolment, interventions, and assessments

Period	Screening	Allocation	Intervention period		Close-out	Follow-up
Time point	0 week	0 week	0 week	1–12 weeks	13 weeks	24 weeks
Inclusion and exclusion criteria	X					
Demographic data	X					
Informed consent	X					
Allocation		X				
LRT-BFR group				X		
CRT group				X		
Control group				X		
Body composition			X		X	X
Muscle strength			X		X	X
Muscle performance			X		X	X
Pulmonary function			X		X	X
Blood biomarkers			X		X	X
CVD risk factors			X		X	X
SF-36			X		X	X
Nutritional intake			X	X	X	X
Patient compliance				X	X	X
Dropout reasons				X	X	X
Adverse events				X	X	X

*CVD* Cardiovascular disease, *SF-36* 36-item Short-Form Health Survey, *LRT-BFR* Low-load resistance training with blood flow restriction, *CRT* Conventional high-load resistance training

### Inclusion criteria

1. Older people aged ≥ 65 years
2. Sarcopenia is diagnosed according to the criteria proposed by the AWGS. Sarcopenia is defined by ASMI < 7 kg/m^2^ for male and < 5.7 kg/m^2^ for female, using a bioelectrical impedance analyzer; 6-m walk (< 1 m/s); and/or handgrip strength (HG, < 28 kg for male and < 18 kg for female)
3. Participants with physical disabilities (such as loss of hands, foot, or limbs) or suffering from dyskinesia disease who are unable to perform measurements and resistance training program
4. Participants with cognitive impairment, dementia, or severe mental illness
5. Not engaging in an exercise program or receiving exercise guidance (especially resistance training)

### Exclusion criteria

1. History of deep venous thrombosis and/or blood clotting disorders
2. Uncontrollable arterial hypertension (blood pressure > 160/100 mmHg) and dyslipidemia (total cholesterol > 220 mg/dl)
3. History of stroke, heart failure, coronary ischemia, coronary arrhythmias, peripheral arterial disease, pulmonary diseases, and musculoskeletal problems
4. Current use of double antiplatelet agents: aspirin, Aggrenox, cilostazol, eptifibatide, ticlopidine, and tirofiban
5. Current use of anticoagulants

### Termination criteria

1. Participants with serious adverse events or complications, who are not enabled to continue the trial
2. Participants withdrew their consent from the trial for any reason
3. Participants lost to follow-up

### Ethics approval

This study was approved by the Ethical Committee of Xinhua Hospital Chongming Branch in November 2020 (approval no. CMEC-2020-KT-42). The study was registered in the Chinese Clinical Trial Registry (ChiCTR2100042803) in January 2021.

Demographic data (sex, height, age, household status, lifestyle habits, blood pressure, physical activity, and history of comorbidities), physical fitness results, questionnaire results, and signed consent forms will be gathered. The electronic data will be stored in an encrypted computer, and paper data will be stored at the project office in Xinhua Hospital Chongming Branch.

The investigators will perform the intervention in accordance with the protocol. Any questions about the protocol will be consulted with the head of Xinhua-Chongming hospital. If it involves modifications made to the protocol, the project leader will convene all research staffs to discuss and decide. Finally, the updated protocol will be reported to the ethics committee of Xinhua-Chongming hospital. All information of this protocol will be recorded on www.chictr.org.cn.

### Recruitment

Subjects will be recruited using three approaches: (1) the annual community physical examination in four communities in the Chongming District, (2) educational lectures and talks about sarcopenia at community centers, and (3) home visits in cooperation with community workers to check on the health of home-dwelling older people who do not participate in community activities regularly. The subjects will be screened on site by SARC-F, a valid screening tool for sarcopenia in Chinese older people [29] that assesses 5 components: strength, assistance in walking, rising from a chair, climbing stairs, and falls. The questionnaire has a score range from 0 (the best) to 10 (the worst) with a SARC-F score ≥ 4 points diagnosed as having a risk of sarcopenia. Subjects with a SARC-F score ≥ 4 points will complete the AWGS screening consisting of assessment of appendicular skeletal muscle mass index (ASMI) with a portable bioelectrical impedance analyzer system (Inbody, S10, Korean); handgrip strength with a hand dynamometer (HK-6000, Hengkang, China); and a 6-m walk to determine walking speed. A physician will conduct the examination and consultation on subjects who meet the inclusion criteria, and excludes subjects who meet the exclusion criteria.

### Randomization and blinding

Participants who meet the inclusion criteria and do not meet the exclusion criteria will be informed about the purpose and procedures of the study, what it will involve for them, and any risks involved in taking part as well as how to obtain more information about the study, low- and high-load resistance training and sarcopenia. After informed consent is obtained, subjects will be randomized into one of three groups: LRT-BFR, CRT, or control at 1:1:1 ratio. The randomization sequence, stratified by sex and age, will be generated and stored by a statistician who is not participating in the intervention and evaluation of the study, using SPSS version 20 statistical package (SPSS, Inc., USA). The random numbers and grouping assignments will be delivered in an encrypted electronic file to a research staff from another research for keeping this information. The therapists of this study will check the file and perform the intervention according to the grouping assignments. Except for therapists, all other participating staff, including the statistician, outcome assessors, and data managers will be blinded to the group assignment.

### Interventions

#### Group protocols

Before the group protocols begin, all subjects will complete a one-repetition maximum (1RM) strength test to assess their muscle strength and to determine the intensity for resistance training in training groups. In addition, they will complete three pre-intervention lessons with the resistance training exercises to familiarize themselves with the training sessions. Subjects randomized to the control group will receive health education sessions only. The researchers will record the attendance of each participant in each training session. And if a participant misses a scheduled training session, the session will be completed within the training week.

#### Resistance training program

Both resistance training intervention groups will receive three weekly resistance training sessions for 12 weeks with training intensities specific to their group assignment (see Table 2). Both groups will use Thera-band elastic bands to deliver the resistance exercises. Both groups will perform upper limb exercises (elbow extension and elbow flexion), followed by lower limb exercises (leg press and knee extension). The resistance training protocol of LRT-BFR will consist of 3 sets of 30-15-15 repetitions of each exercise, performed at an increased intensity from 20 to 30% 1RM by different Thera-band elastic bands. Subjects will have a 20-s rest interval between sets and a 30-s rest interval between exercises. The CRT group will perform 3 sets of 15 repetitions at 60% 1RM in the first 4 weeks, 3 sets of 12 repetitions at 65% 1RM in the second 4 weeks, and 3 sets of 10 repetitions at 70% 1RM in the third 4 weeks. They will have a 60-s rest interval between sets. The progressive resistance is also achieved by using Thera-band elastic bands with different tensions.

**Table 2:** Details of the training protocol in the intervention groups

Weeks	Group	Sets	Repetitions	Intensity (%1RM)	Frequency (times/week)	Exercises	Interval between sets (s)
1–4	LRT-BFR	3	30, 15, 15	20	3	Elbow extension, elbow flexion, leg press, knee extension	30
	CRT	3	15	60	3		60
5–8	LRT-BFR	3	30, 15, 15	25	3	Elbow extension, elbow flexion, leg press, knee extension	30
	CRT	3	12	65	3		60
8–12	LRT-BFR	3	30, 15, 15	30	3	Elbow extension, elbow flexion, leg press, knee extension	30
	CRT	3	10	70	3		60

*LRT-BFR* Low-load resistance training with blood flow restriction, *CRT* Conventional high-load resistance training

#### Blood flow restriction

In the LRT-BFR group, blood flow restriction will be stimulated by inflating nylon cuffs connected to an inflator (TD312 Hokanson^TM^, Bellevue, USA) to the pressure as 50% of limb occlusion pressure (LOP). In order to provide personalized cuff pressure (mm Hg), technicians will measure the LOP of lower and upper limbs for each subject before the exercise intervention with a vascular Doppler probe (DV-600, Marted, São Paulo, Brazil). The assessment method of LOP will be as follows: while subjects are reclining, an ultrasound probe will be placed over the tibial and radial arteries to capture their auscultatory pulse in the lower and upper limbs, respectively. Remaining supine, the cuff will be placed at the most proximal part of the subject’s thigh for the lower limb resistance and at the most proximal part of the arm for the upper limb resistance. The cuff will be inflated to the point in which the auscultatory pulse is interrupted. The cuff pressure exerted at this time is referred to as the LOP. At this point, the subjects will then begin the LRT resistance training session. The exerted cuff pressure should not cause the subjects to feel pain or any discomfort during training and the pressure can be adjusted as needed according to the subject’s comfort. The cuff will remain inflated from the beginning to the end of each training session, including the rest intervals, and will be deflated immediately after the end of the last set.

#### Control group education sessions

The control group will meet once a week at the community center and receive a 60-min educational session on aging and health (see Table 3). Subjects will be instructed to maintain their usual lifestyles, and while some subjects may engage in some form of physical activity, they will not be provided an exercise program nor will they be encouraged to participate in a physical activity involving resistance training. To assess the subject’s usual physical activity levels, the Chinese version of the short form of the International Physical Activity Questionnaire (IPAQ) will be administered every week. The seven items in the short IPAQ provide an assessment of the duration and frequency of walking and moderate- and vigorous-intensity physical activity per week. The short IPAQ is scored by multiplying the duration in minutes per day, frequency in days per week, and a MET value of each activity (walking 3.3 METS, moderate-intensity 4.0 METS, vigorous-intensity 8.0 METS) to create MET-minute scores. The MET-minute scores are summed across activities to create a total MET-minute per week score for walking and moderate- and vigorous-intensity activities.

**Table 3:** Details of education session topic in the control group

Week	Group	Education session topic	Frequency (times/week)
1–4	CG	Geriatric diseases and disease risk reduction	1
5–8	CG	Nutrition and physical activity for older people	1
9–12	CG	The importance of social support and care from friends, families, and communities	1

*CG* Control group

#### Nutritional intake

All the participants will maintain normal dietary habits during the 12-week intervention and follow-up period. They will record all their nutritional intakes including the amount of protein they took by 3-day dietary record (two weekdays and one weekend day in a week) per week on the notebook [30]. The weekly recording work is assisted and supervised by community works.

### Outcome measurements

The following measures will be conducted at baseline (0 week), after the 12-week intervention (12 weeks), and at follow-up (24 weeks).

#### Primary outcomes

##### Muscle strength in the lower limb

As the standard 1RM tests may be too difficult and induce skeletal muscle injury in older people with sarcopenia [31], the lower limb muscle strength will be assessed by an estimated 1RM of knee extension [32]. The estimated 1RM is calculated as follows: estimated 1RM (kg) = submaximal weight (kg)/(1.0278 − 0.0278 × maximal number of repetitions) [33]. According to the measurement method in the study of Brzycki et al. [31], the test is performed by subjects sitting on an extensor chair with a weight plate in kilograms [34]. The initial weight plate load is set at 45% of the body mass for female and 64% for male [35]. If they can repeat more than 10 times, they will be given a heavier weight to test. When the maximum number of repetitions is 10 or less, the weight is considered to be the submaximal weight. The maximal number of repetitions performed and submaximal weight will be used to calculate the estimated 1RM.

#### Secondary outcomes

##### Body composition

A bioelectrical impedance analyzer (Inbody S10, Korean) will be employed to measure body weight (BW), skeletal muscle mass (SMM), appendicular skeletal muscle mass (ASM), body fat mass (BFM), skeletal muscle mass index (SMI), and appendicular skeletal muscle mass index (ASMI). Of these indicators, the first four measures (BW, SMM, ASM, and BFM) are obtained directly through the test. The latter two indicators (SMI, ASMI) are obtained through calculation. According to methods described previously, SMI will be calculated as an SMI divided by the squared height (SMI = SMM/height^2^, kg/m^2^), and ASMI will be calculated as an ASM divided by the squared height (ASMI = ASM/height^2^, kg/m^2^) [1, 36].

##### Handgrip strength

The upper limb muscle strength will be assessed by the handgrip strength (HG) on each hand using a hand dynamometer (HK-6000, Hengkang, China). Subjects will be instructed to hold the dynamometer in a standing position, with the upper limb abducted at a 30-degree angle from the body. The maximum handgrip strength will be measured 3 times sequentially on each hand with the highest value in kilograms recorded [37].

##### Muscle performance

Muscle performance will be evaluated using the Short Physical Performance Battery (SPPB) test and the 6-m walk test. The SBBP incorporates performance in three tests: a standing balance test, a gait speed test, and a chair sit-stand test [38]. In the standing balance test, subjects will be required to stand and remain in three increasingly difficult positions (side-by-side, semi-tandem, and full-tandem stances) for 10 s each. In the gait speed test, the subjects will walk 8-feet at a normal pace for two times with the faster of two walks recorded. In the chair sit-stand test, the subjects will fold their arms across their chest and stand up and sit down five times as quickly as possible. The test will be timed from the starting sitting position to the final standing position. The score for each test ranges from 0 (unable to complete the test) to 4 (complete the test with no difficulty). The scores of the three tests will be summed with the highest total score of 12. For older people, SPPB scores are interpreted as follows: 10–12 = high performance, 7–9 = intermediate performance, and 0–6 = low performance [39]. The 6-m walk test requires subjects to walk at their usual speed for 6 m. Speed will be recorded with a timing device to record the total time it takes to walk 6 m at a usual speed. The test will be performed twice with the average of tests recorded [40].

##### Pulmonary function

Pulmonary function tests (forced vital capacity [FVC], peak expiratory flow [PEF], maximal inspiratory pressure [MIP], and maximal expiratory pressure [MEP]) will be conducted on a spirometer (Quark PFT ERGO, Italy) according to the standards of the American Thoracic Society (ATS) and European Respiratory Society (ERS) [41]. Each subject will be tested three times and the maximum value will be recorded.

##### Blood biomarkers and CVD risk factors

Following an overnight fast, study nurses will collect blood from the antecubital vein 48 h before the beginning of the 12-week intervention, 7 days after the end of the intervention, and 12 weeks after the end of the intervention, respectively. Enzyme-linked immunoassay (ELISA) will be used to determine the level of inflammatory biomarkers (IL-6, CRP, TNF-α), hormone (GH, IGF-1), growth factors (FST, MSTN), and CVD risk factors (fasting plasma glucose [FPG], total cholesterol [TC], triglycerides [TG], high-density lipoprotein cholesterol [HDL-C], and low-density lipoprotein cholesterol [LDL-C]). In addition, CVD risk factors also include systolic blood pressure and diastolic blood pressure.

##### Health-related quality of life

Health-related quality of life will be assessed using the validated Chinese version of the 36-item Short-Form Health Survey (SF-36). The SF-36 consists of 36 items, and it is a brief self-administered questionnaire designed to assess functioning and well-being in adults on eight health scales: physical functioning, role-physical, bodily pain, general health, vitality, social functioning, role-emotional, mental health, and reported health transition [42]. The score ranges from 0 to 100 with higher scores reflecting a better health-related quality of life.

### Sample size

The sample size was calculated with PASS (version 11.0). According to a previous study, lower limb muscle strength is the most relevant outcome to show the efficacy of blood flow restriction. Thus, it was used as the primary outcome for calculating sample size [43, 44]. According to the data used in the study by Brandner et al. [44], the expected mean changes in lower limb muscle strength were approximately 11% for the BFR training group, 16% for high-load resistance training, and 0% for the control group with a pooled standard deviation of 13%. This sets the estimated total sample size at 42 subjects (14 in each group) to meet 80% power and an alpha of 0.05. Considering the frailty of some older people with sarcopenia, up to a 20% dropout rate is expected. Therefore, the sample size is set at 51 subjects (17 in each group).

### Statistical analysis

The clinical and questionnaire results will be collected in this study. Among these results, results from the participants both seven clinical evaluations and one questionnaire will all be included in the data analysis. SPSS version 20 statistical package (SPSS, Inc., USA) will be used to analyze all data with *p* < 0.05 indicating statistical differences. Data will be analyzed for all participants in an intent-to-treat plan. Continuous data will be expressed as mean ± standard deviation (M ± SD), or median. For between-group comparisons of baseline characteristics, one-way analysis of variance (ANOVA) or Kruskal-Wallis tests will be used for parametric and non-parametric continuous data, respectively. The chi-square test will be used for categorical data.

According to the intention-to-treat principle, a linear mixed model will be used for analyzing the differences between the three interventions group over time on the outcome variables. Missing data will be assumed missing at random and handle by a linear mixed model by using maximum likelihood.

### Safety monitoring

The training intervention will be carried out under the supervision of researchers and trainers. The adverse events (AEs) and serious adverse events (SAEs) will be recorded for all participants during each training session. SAEs will be reported to the sponsor and principal investigator to find a reasonable solution. If necessary, medical services should be provided as compensation to those who suffer harm from trial participation. Subjects have the right to withdraw their consent to participate in the study at any time for any reason, without any consequences for further treatment.

### Data management and monitoring

All study information will be stored in Microsoft Excel 365 by a data manager using an electronic database. The database will record all subject data to include the baseline characteristics, pre- and post-assessments, and possible adverse events. The computer and database will use password protection with the database accessible to the study researchers only. To ensure the confidentiality of the data, all subjects will be provided with identification numbers. All researchers will have access to the final trial data. Electronic data and paper documents will be kept for 5 years, after which they will be destroyed. Data Monitoring Committee (DMC), which is independent from the sponsor and competing interests, comprises clinical experts, trial experts, investigators, and a statistician. It will conduct regular monitoring tests to ensure the authenticity of the data.

As for the audition, the project organization will hold a meeting every 6 months to review and evaluate the trial progress. The process will be independent from investigators and the sponsor.

### Dissemination

The results of this study will be published in peer-reviewed journals and be presented at domestic or international academic congresses. After being published, the individual results will be reported by the researcher to the participants of this study. Any changes or additions to the protocol will be documented at www.chictr.org.cn.

## Discussion

To the best of our knowledge, this study is the first RCT that will systematically measure and compare the efficacy and safety of LRT-BFR and CRT with a control group on muscle strength, body composition, muscle performance, pulmonary function, blood biomarkers, CVD risk factors, and quality of life in older people with sarcopenia. An increasing number of studies have shown that sarcopenia is associated with several adverse health outcomes such as falls and fractures [3] and that sarcopenia is strongly associated with disability and premature mortality in older people [45]. Thus, prevention and treatment of sarcopenia should be highly valued by society and clinical staff.

Prior research to increase muscle mass has relied on CRT protocols designed to stress the skeletal and muscular system for optimal strength gain [46]. In search of a less intense method to increase muscle mass in older persons, LRT-BFR has been explored for its efficacy and safety [25, 47]. Studies have focused on measuring muscle mass and strength and have explored the role of blood biomarkers to explain physiological mechanisms. The studies show that LRT-BFR can increase muscle size and muscle strength by promoting hormone secretion (GH [48] and IGF-1 [49]) and protein synthesis (mammalian target of rapamycin [mTOR]) [50] and decreasing MSTN [51]. However, as noted earlier, most of these studies have been completed in athletes and healthy young people, not older people with sarcopenia. Given that aging weakens the benefits of exercise [45], it is important to determine the efficacy of LRT-BFR on clinical outcomes and blood biomarkers in older people with sarcopenia. As blood biomarkers have been shown to be associated with the development of sarcopenia, this study will measure common blood biomarkers of IL-6, TNF-α, CRP, GH, IGF-1, and MSTN and FST as outcome measures. Changes in these blood biomarkers from pre-to-post LRT-BFR may reflect the efficacy and mechanisms of LRT-BFR on the development of sarcopenia. In addition, this study also evaluates the efficacy of LRT-BFR on CVD risk factors to discuss the potential implication of LRT-BFR on cardiovascular health.

Compared with previous studies, this study has the following strengths: First, the study will use elastic bands as the resistance training mode to reduce the impact of injuries. Kerr et al. [15] showed that people aged > 55 years are more likely to suffer injuries with weight machines than those aged < 54 years. They believed that this may be because the sense of security brought by the weight machine may cause people to exceed their capacity in resistance training. Elastic band training programs are safe and simple for older people to perform and they can be easily accessed and conducted in community centers. Second, in previous studies, the LRT-BFR has been conducted on the lower limbs primarily. As such, the evaluation is limited to the muscle mass and muscle strength of specific limbs to include the cross-sectional area of the quadriceps muscle and lower limb muscle strength [23, 52, 53]. Sarcopenia is a syndrome characterized by loss of whole-body muscle mass, and it is an important factor in the decrease of peripheral and respiratory muscle strength among older people [54].

As the elastic component and parenchymal tissue of the lung degenerate with age, the compliance of the chest wall is reduced, and the intercostal muscle mass and strength decrease. These changes are accompanied by a decrease in pulmonary function [55]. A study indicated that low pulmonary function among community-dwelling older people was found to be related to low muscle mass [56]. Another study showed that older people with sarcopenia had respiratory muscle weakness symptom when compared to non-sarcopenic older people [57]. Previous studies had focused on peripheral muscle strength, and there is a lack of studies to demonstrate the association between resistance training and respiratory muscle strength on sarcopenia among community-dwelling older people. Thus, this study will employ resistance training for the whole-body muscle using elastic bands and will measure changes in the upper and lower limb muscle mass and peripheral and respiratory muscle strength. Third, the study will measure blood biomarkers before and after LRT-BFR training to explore physiological mechanisms of sarcopenia in older people with sarcopenia. As little is known about how LRT-BFR can improve muscle mass and strength, this research can improve the understanding of the control and treatment of sarcopenia.

The study also has some limitations. As a new training method, there are few studies that report the safety and potential adverse outcomes of LRT-BFR in older people, especially those with sarcopenia. Cook et al. [58] noted that the fatigue caused by LRT-BFR is similar or may even be higher than that caused by high-load resistance training. Although fatigue may have the potential to stimulate muscle growth, it can also reduce the compliance of resistance training in older people, especially those who are frail. Therefore, the fatigue level of subjects will be monitored closely during and after the training sessions with sessions modified as needed.

To date, although sarcopenia affects a large proportion of the population in the world, there are no pharmacologic agents to prevent or reduce its impact. Nor is there a consensus on the prevention and treatment of sarcopenia. Accordingly, the management of sarcopenia is still an unknown field. As a novel training method, LRT-BFR has the potential to make it easier for older people with sarcopenia to complete exercise training and achieve the same training effects as CRT in muscle mass and strength without increasing the risks of injury. Therefore, as a feasible alternative training method to CRT, LRT-BFR may be a potential training method to prevent the progression of sarcopenia.

## Trial status

The study was registered with the Chinese Clinical Trial Register (ChiCTR2100042803) on 28 January 2021. The subject recruiting was started on 1 May 2021. Recruitment is expected to end around June 2021.

## Supplementary Information

**Additional file 1.** SPIRIT Checklist for *Trials*: Recommended items to address in a clinical trial protocol and related documents.

## Data Availability

Not applicable.

## Abbreviations

CRT: High-load resistance training
LRT-BFR: Low-load resistance training combined with blood flow restriction
IL-6: Interleukin-6
TNF-α: Tumor necrosis factor alpha
CRP: Serum C-reactive protein
GH: Growth hormone
IGF-1: Insulin-like growth factor 1
MSTN: Myostatin
FST: Follistatin
FPG: Fasting plasma glucose
TC: Total cholesterol
TG: Triglycerides
HDL-C: High-density lipoprotein cholesterol
LDL-C: Low-density lipoprotein cholesterol
BW: Body weight
SMM: Skeletal muscle mass
ASM: Appendicular skeletal muscle mass
ASMI: Appendicular skeletal muscle mass index
BFM: Body fat mass
SMI: Skeletal muscle mass index
HG: Handgrip strength
1RM: One-repetition maximum
LOP: Limb occlusion pressure
IPAQ: International Physical Activity Questionnaire
SPPB: Short Physical Performance Battery test
FVC: Forced vital capacity
PEF: Peak expiratory flow
MIP: Maximal inspiratory pressure
MEP: Maximal expiratory pressure
SF-36: 36-item Short-Form Health Survey
